# Plasma levels of tissue inhibitor of metalloproteinase-1 in patients with type 1 diabetes mellitus associate with early diabetic neuropathy and nephropathy

**DOI:** 10.1177/14791641211002470

**Published:** 2021-03-27

**Authors:** Nektaria Papadopoulou-Marketou, Per A Whiss, Andreas C Eriksson, Lars Hyllienmark, Ioannis Papassotiriou, Jeanette Wahlberg

**Affiliations:** 1 Division of Diagnostics and Specialist Medicine, Department of Health, Medicine and Caring Sciences (HMV), Linköping University, Linköping, Sweden; 2 University Research Institute of Maternal and Child Health and Precision Medicine, National and Kapodistrian University of Athens, Medical School, Aghia Sophia Children’s Hospital, Athens, Greece; 3 Division of Drug Research, Department of Biomedical and Clinical Sciences (BKV), Linköping University, Linköping, Sweden; 4 Clinical Neurophysiology, Karolinska University Hospital, Stockholm, Sweden; 5 Department of Clinical Biochemistry, Aghia Sophia Children’s Hospital, Athens, Greece

**Keywords:** Type 1 diabetes, diabetic neuropathy, diabetic nephropathy, biomarkers, microvascular complications, TIMP-1

## Abstract

**Background:**

Tissue inhibitor of metalloproteinase-1 (TIMP-1) has been suggested as a marker for abnormal regulation of tissue remodelling in type 1 diabetes. Metalloproteinase-9 (MMP-9) has been associated with matrix turnover, and Neutrophil gelatinase associated lipocalin (NGAL) is a marker of tubular injury in diabetic nephropathy. The aim was to analyse these biomarkers to unmask early diabetic complications.

**Methods:**

Thirty-three type 1 diabetes patients, aged 20–35 years, and disease duration 20 ± 5.3 years were included. Along with clinical examination, neurophysiological measurements, routine biochemistry, plasma concentrations of TIMP-1, MMP-9 and NGAL were determined with immunoenzymatic techniques.

**Results:**

TIMP-1 correlated with abnormal unilateral and bilateral vibratory sense foot perception (*r* = −0.49 and *r* = −0.51, respectively), foot neuropathy impairment assessment score (NIA; *r* = −0.55), neuropathy symptom assessment score (*r* = 0.42), microalbuminuria (*r* = 0.50) and eGFR (*r* = −0.45). MMP-9 correlated with impaired foot NIA (*r* = 0.51). Multiple regression analysis showed an association for TIMP-1 (*p* = 0.004) with impaired neurophysiological examinations and renal dysfunction along with NGAL (*p* = 0.016 and *p* = 0.015 respectively).

**Conclusions:**

This study suggests that plasma levels of TIMP-1, MMP-9 and NGAL may serve as useful biomarkers in unravelling subclinical neuropathy and nephropathy in type 1 diabetes.

## Introduction

Type 1 diabetes is linked to an increased risk of macro- and microvascular complications but the leading mechanisms have not been fully established.^1–3^

Peripheral diabetes neuropathy is a common microvascular complication of type 1 diabetes, which increases in frequency with the duration of disease and its progression is associated with a poor metabolic control. Abnormal nerve conduction tests may be the first objective quantitative indication of the condition.^4^ It was previously suggested that electrophysiological abnormalities seen early in asymptomatic people with type 1 diabetes might predict clinical neuropathy later on.^4–6^

Earlier studies have provided evidence that nephropathy in subjects with type 1 diabetes is associated with generalised endotheliopathy and cardiovascular disease^7^ while the pathophysiologic changes that lead to renal function decline are associated with structural injury in both the glomerular and tubulointerstitial compartments.^3^

It has been suggested that impaired regulation of extracellular matrix (ECM) remodelling by matrix metalloproteinases (MMPs) is associated with development of vascular damage.^8^ Increased MMP activity has been correlated with increased matrix turnover in atherosclerosis, especially in diabetic nephropathy, with glomerular basement membrane thickening.^9^

MMPs are zinc- and calcium-dependent endopeptidases, able to degrade and rebuild proteins like collagen, elastin, gelatine and casein.^10^ Increased plasma levels of MMPs have been observed in people with type 1 diabetes, while increased levels of serum MMP-9, as well as increased MMP-9-to-Tissue inhibitor of metalloproteinase (TIMP) ratio, have been related to retinopathy.^11^ Several studies have previously reported that hyperglycemia, low-grade inflammation and endothelial dysfunction have been associated with higher plasma and tissue values of MMPs and TIMP in type 1 diabetes.^10^ In addition, aldosterone modulates metalloproteinase-9 (MMP-9) and MMP-9/neutrophil gelatinase-associated lipocalin (NGAL) protein complex in neutrophils.^12^ Previous studies showed that aldosterone up-regulated proMMP-9, active MMP-9 protein release and the MMP-9/NGAL protein complex, known as a main mediator of plaque instability and vascular remodelling.^13^ TIMP-1 inhibits the action of MMP-9^14^ and an unfavourable balance of the two proteins has been linked to pathological conditions such as arthritis, tumorigenesis and metastatic disease, neurodegenerative disorders, atherosclerosis and fibrosis.^15^ TIMP-1 was recently suggested as a marker for abnormal regulation of matrix remodelling after a cardiovascular event in individuals with type 1 diabetes, as well as macroalbuminuria and advanced renal fibrosis in early diabetic nephropathy.^10^ NGAL serum values associate with renal function decline as well as increased systolic blood pressure in nonalbuminuric children and adolescents with type 1 diabetes, suggesting this marker as useful diagnostic tool in prediction of diabetic kidney disease (DKD) progress.^16^ Thus, another study reported that NGAL was independently associated with all-cause and cardiovascular disease mortality in a U-shaped mode as well as a highly significant nonlinear correlation of NGAL with creatinine, cystatin C and estimated glomerular filtration rate (eGFR).^17^

Given these considerations, we hypothesised that high plasma levels of MMP-9 and its inhibitor TIMP-1 as well as NGAL can be related to chronic microvascular complications in individuals with type 1 diabetes, such as diabetes nephropathy and diabetes neuropathy.

## Materials and methods

The study group consisted of thirty-three patients (17 male and 16 female) with type 1 diabetes, 20–35 years of age and disease duration 20 ± 5.3 years. All patients were recruited at the Department of Endocrinology at the University Hospital of Linköping, Sweden. The study protocol was approved by the Local Research Ethics Committee, Linköping and informed consent was obtained by all the participants. The study was designed as cross-sectional with extended clinical and laboratory examination of the patients at scheduled hospital visits. All participants who were examined on at least one previous occasion with nerve conduction tests were included in the current study. All patients were receiving intensive insulin therapy from disease onset.

The diagnosis of type 1 diabetes was based on the presence of the positive titre of at least one of the autoantibodies related to type 1 diabetes mellitus (glutamic acid decarboxylase autoantibodies (GADA), islet antigen 2 antibody 2A (IA2A), islet cell antibodies (ICA)).

At the time of evaluation, no participant had a history of neurological or metabolic disease other than type 1 diabetes. In addition, no participant had a history of alcohol abuse, or of pharmacological therapy known to have an adverse effect on peripheral nerve function. A direct query, modified from Dyck et al.^5^ was previously suggested for typical symptoms of neuropathy. A clinical examination of the tendon reflexes was done bilaterally in the quadriceps and gastrocnemius, while the vibratory sense was tested at first metatarsal bilaterally with a 128-Hz tuning fork.

The neurophysiological examination included bilateral measurements of both peroneal and median motor nerve conduction velocity (MCV), compound muscle action potential (CMAP) amplitude, sensory nerve action potential (SNAP), sural and median sensory nerve conduction velocity (SCV). All amplitudes (i.e. CMAPs, SNAPs) were measured from peak to peak, and sensory nerves were studied with orthodromic recording of SNAPs. The results of the examination regarding neurophysiological tests have been published earlier as well as a description of the controls (*n* = 128).^4,18,19^

Clinical manifestations or symptoms with numbness, paraesthesia or pain in either the lower or the upper extremities were summed up to assess a neuropathy symptom assessment score denoted as neuropathy impairment assessment (NIA).^4^ NIA served as an assessment tool of sensory screening for touch, pinprick, vibration (Vibrameter; Somedic, Stockholm, Sweden) and temperature (Marstock technique)^20^ measured on the first metatarsal, tibial and dorsum of the feet. NIA results were graded as normal, decreased, or absent.

Diabetic nephropathy was assessed by examining albumin excretion rates from duplicate 24-h urine collections.^21^ Micro- and macroalbuminuria were defined as an albumin excretion rate between 30 and 300 mg/24 h, or above 300 mg/24 h, respectively. Estimated glomerular filtration rate (eGFR) was calculated using the CKD-EPI formula.^22^ Furthermore, Cystatin C concentration was measured in heparinised plasma by an immunonephelometric technique using the BN Prospec nephelometer (Siemens Healthcare Diagnostics, Erlangen, Germany). The interassay coefficient of variation (CV) of the assay was 5.05%, and 4.86% at mean concentrations of 0.97 and 1.91 mg/L, respectively and the reference range in plasma was 0.47–1.09 mg/L. Apart from routine biochemical markers, Matrix Metalloproteinase-9 (MMP-9) and its tissue inhibitor TIMP-1 were analysed in heparinised plasma using commercially available enzyme immunoassays provided by R&D Systems (Minneapolis, MN, USA). The intra-assays and inter-assays CVs ranged between 2.0% and 2.9% and between 6.9% and 7.9% for MMP-9, between 4.2% and 5.0% and between 3.9% and 4.9% for TIMP-1 respectively. Serum Neutrophil gelatinase associated lipocalin (NGAL) was measured by means of immuno-enzymatic assay (Bioporto, Denmark). The intra-assays and inter-assays CVs ranged between 10% and 11% and between 4% and 14%, respectively.

## Statistical analysis

Data are presented as mean values and statistical significance was considered at *p* < 0.05 and 95% confidence interval (CI) for the correlation coefficient. All the statistical procedures were performed using the MedCalc Statistical Software version 17.2 (Ostend, Belgium). Pearson parametric correlation analysis was used to determine whether the values between two variables were associated. Multiple regression analysis was used to examine the association between one dependent variable and at least one independent variable.

## Results

TIMP-1 in plasma from patients with type 1 diabetes had a mean value of 91 ng/mL (95% CI: 85–97). Levels of TIMP-1 in subjectively healthy controls earlier reported from our department are 92 ng/mL (range 80–103).^23^ MMP-9 had a mean value of 82 ng/mL in the type 1 diabetes patients (95% CI: 64–99) as compared to 53 ng/mL (range 43–85), in healthy controls.^23^ Patient characteristics and values for all analysed plasma markers are shown in Table 1 and pathological complications and presence of diabetic neuropathy are shown in Table 2.

**Table 1.** Patient characteristics and laboratory data (*n* = 33).

	Mean	95% CI	Minimum	Maximum
Age (years)	28.0	27.0–29.0	20.0	35.0
Duration of type 1 diabetes (years)	20.1	18.7–21.5	10.0	31.0
Systolic arterial pressure	123.9	121.4–126.3	108.0	140.0
Diastolic arterial pressure	77.9	75.8–80.0	60.0	98.0
BMI	26.2	25.1–27.4	20.1	40.4
Cystatin C (mg/L)	0.72	0.67–0.76	0.39	1.30
eGFR (mL/min/1.73 m^2^)	122.25	113.06–131.44	56.60	234.30
HbA1c (%)	7.47	7.06–7.88	4.60	11.7
MMP-9 (ng/mL)	81.99	64.60–99.38	24.20	290.76
MMP-9/TIMP1 ratio	0.90	0.71–1.09	0.29	3.15
NGAL (ng/mL)	86.32	78.23–94.41	39.70	186.50
TIMP-1 (ng/mL)	91.21	85.26–97.16	67.48	148.20

CI: confidence interval; BMI: body mass index; eGFR: estimated glomerular filtration rate; MMP-9: matrix metalloproteinase-9; TIMP1: tissue inhibitor of metalloprotein 1; NGAL: neutrophil gelatinase-associated lipocalin.

**Table 2.** Patient pathological complications and abnormalities (*n* = 33).

	Normal	Pathological complications and abnormalities
Micro- or macroalbuminuria	*n* = 29	*n* = 4
Retinopathy	*n* = 29	*n* = 4
Neuropathy symptom assessment score	*n* = 26	*n* = 7
Suralis sensory nerve action potential	*n* = 28	*n* = 5
Peroneal compound muscle action potential amplitude	*n* = 31	*n* = 2
Peroneal motor nerve conduction velocity	*n* = 19	*n* = 14
Sural sensory nerve conduction velocity	*n* = 26	*n* = 7

**Figure 1.**
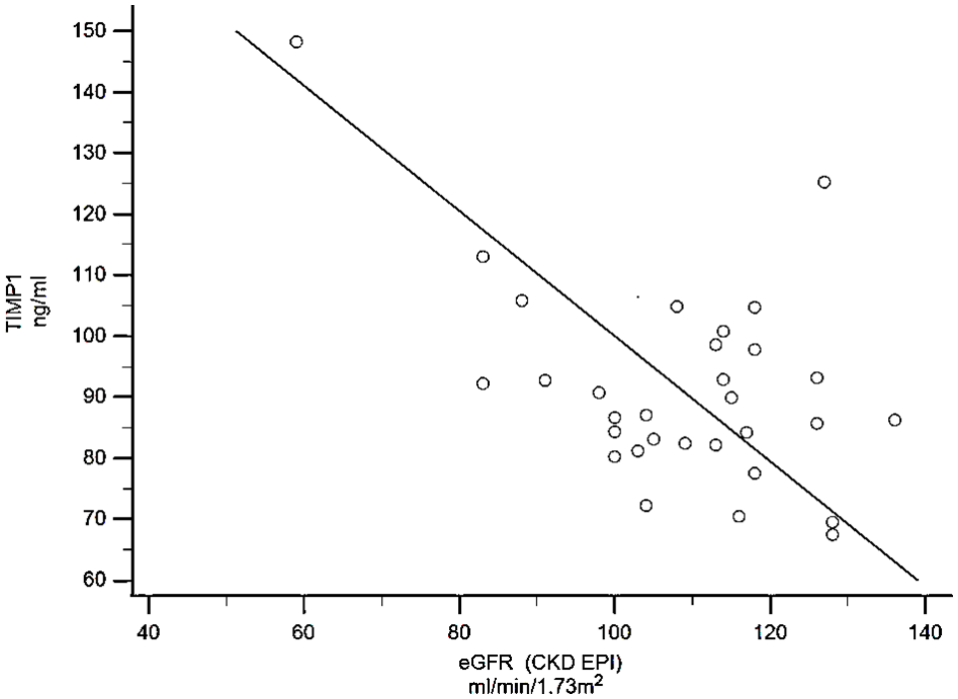
TIMP-1 showed a negative correlation with estimated glomerular filtration rate (eGFR) in young adults with type 1 diabetes (*r* = −0.45, *p* = 0.008, 95% CI = −0.68 to −0.12, *n* = 33).

TIMP-1 value showed a positive association with impaired renal function assessed by increased cystatin C, NGAL and microalbuminuria, as well as decreased eGFR (Table 3, and exemplified with eGFR in Figure 1). Microalbuminuria was observed in 3 out of 33 patients and macroalbuminuria was found in 1 patient. Regression analysis adjusted for microalbuminuria, showed that TIMP-1 correlated positively with NGAL (*p* = 0.011), Cystatin C (*p* = 0.010) and negatively with eGFR (*p* = 0.003).

**Table 3.** Relationship by Pearson correlation coefficient (Pearson *r*) between MMP-9 and TIMP-1 in plasma and other laboratory data and neurophysiological tests in young adults with type 1 diabetes (*n* = 33).

	MMP-9		TIMP-1		NGAL (ng/mL)	
	Pearson *r*	*p* value	Pearson *r*	*p* value	Pearson *r*	*p* value
Cystatin C	−0.16	0.375	0.51	0.002	0.35	0.009
Microalbuminuria	0.39	0.080	0.50	0.007	0.25	0.140
Estimated glomerular filtration rate (eGFR)	0.15	0.410	−0.45	0.008	−0.30	0.028
Neutrophil gelatinase associated lipocalin (NGAL)	0.26	0.014	0.37	0.034	–	–
Unillateral vibratory sense foot perception	0.55	<0.001	−0.49	0.005	0.34	0.019
Bilateral vibratory sense foot perception	0.29	0.114	−0.51	0.004	0.41	0.003
Tibial neuropathy impairment assessment (NIA; total score for heat, cold, vibration, touch and pinprick perception)	0.29	0.046	−0.47	0.006	0.18	0.195
Foot neuropathy impairment assessment (NIA; total score for heat, cold, vibration, touch and pinprick perception)	0.51	0.003	−0.55	<0.001	0.15	0.281
Neuropathy symptom assessment score	0.13	0.467	0.42	0.005	0.06	0.679
Symptomatology of allodynia	0.11	0.516	0.58	<0.001	0.29	0.029
Peroneal sensory nerve action potential (SNAP)	−0.19	0.284	−0.39	0.022	−0.21	0.120
Suralis sensory nerve action potential (SNAP)	−0.37	0.033	−0.43	<0.001	−0.18	0.183
Peroneal compound muscle action potential amplitude	−0.19	0.284	−0.42	0.013	−0.21	0.120
Peroneal motor nerve conduction velocity	−0.32	0.063	−0.42	0.014	−0.27	0.048
Suralis motor nerve conduction velocity	−0.13	0.464	−0.49	0.003	−0.33	0.016
Standard deviation scores of bilateral measurements of the sural sensory nerve conduction velocity	−0.13	0.470	−0.65	<0.001	−0.31	0.023

MMP-9: matrix metalloproteinase-9; NGAL: neutrophil gelatinase-associated lipocalin; TIMP-1: tissue inhibitor of metalloprotein-1.

**Figure 2.**
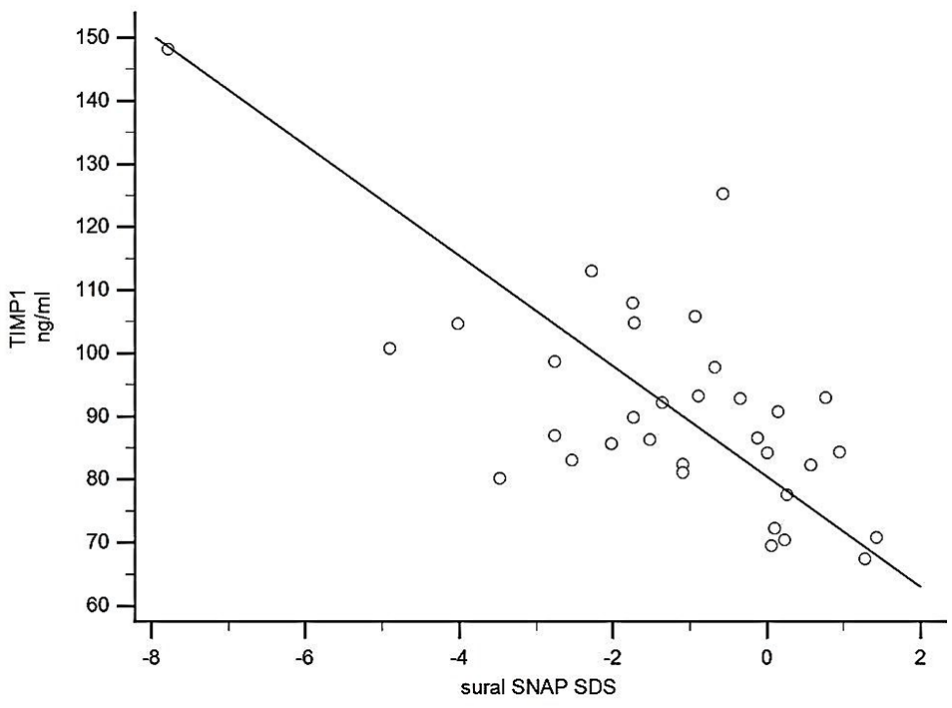
TIMP-1 values correlated negatively with suralis sensory nerve action potential (SNAP) in young adults with type 1 diabetes (*r* = −0.43, *p* < 0.001, 95% CI = −0.81 to −0.41, *n* = 32).

**Figure 3.**
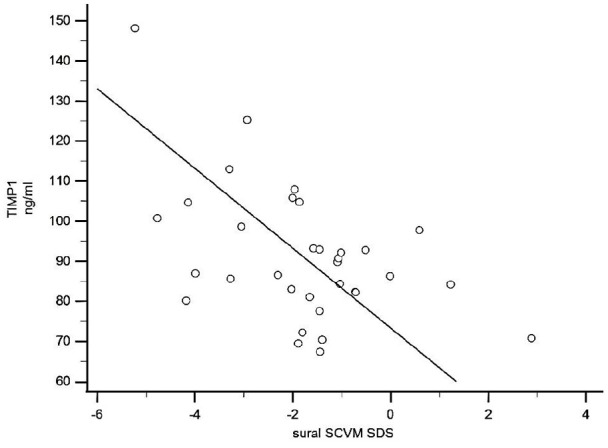
TIMP-1 values correlated negatively with standard deviation scores of bilateral measurements of the sural sensory nerve conduction velocity (SCV) in young adults with type 1 diabetes (*r* = −0.65, *p* < 0.001, 95% CI = −0.80 to −0.40, *n* = 33).

A negative association of TIMP-1 was also evident with several neurophysiological tests indicating neuropathy. As shown in Table 3 plasma levels of TIMP-1 correlated with abnormal vibratory sense foot perception, neuropathy symptoms, impaired sensory nerve action potential, impaired sensory nerve conduction velocity, compound muscle action potential amplitude and motor nerve conduction velocity. Representative examples of these relationships are shown in Figures 2 and 3.

MMP-9 levels correlated with neuropathy impairment assessment regarding foot heat perception as well as impaired vibratory sense and suralis sensory nerve action potential (Table 3). No statistically significant correlation was found between MMP-9 and renal impairment indices.

Calculation of MMP-9 to TIMP-1 ratio values (MMP-9/TIMP-1) revealed no correlations with any of the measurements (data not shown).

TIMP-1 was the only marker that showed significant correlation with all other laboratory data and tests, and unilateral vibratory sense foot perception was the only test that were associated with all biomarkers (Table 3).

Multiple regression analysis regarding impaired neuropathy symptom assessment score showed a statistically significant association with TIMP-1 (*p* = 0.004). Multiple regression analysis for impaired SNAP in sural nerve and CAMP in peroneal nerve demonstrated an association with TIMP-1 (*p* = 0.019 and *p* = 0.030, respectively).

Multiple regression analysis regarding renal impairment showed a statistically significant association with both TIMP-1 and NGAL (*p* = 0.016 and *p* = 0.015 respectively).

## Discussion

This is the first study that assessed associations of MMP-9 and TIMP-1 with long-term microvascular complications in type 1 diabetes, primary diabetic neuropathy, and diabetic nephropathy. The results revealed correlations between these biomarkers and either microalbuminuria or reduced eGFR or neurological impairment. Past studies have shown an association between proinflammatory cytokines such as IL-1β and TNF-α and virus infected or immune activated mononuclear phagocytes with up-regulated TIMP-1.^15^ Several lines of evidence have shown that TIMP-1 may serve as a biomarker of ongoing adverse cardiac remodelling^10^ as changes in the plasma levels of TIMP-1 according to several large-scale cardiovascular outcome studies have been associated with increased mortality. Increased MMP-9 expression/activity has been associated with different stages of inflammation while MMP-9 upregulation has been linked to inflammation in muscular dystrophies and inflammatory myopathies.^23,24^ Besides, it was previously shown that inhibition of MMP activity shortly after sciatic nerve crush helps nerve regrowth by enhancing the rate of Schwann cell mitosis and in a post-damage phase an excellent balance between MMP-9 and TIMP-1 is a critical parameter of the signalling system in the damaged nerve, by regulating Schwann cell mitogenesis and maturation.^25^ However, at the tissue level, MMP-9 is not always hindered by TIMP-1. Thus, neutrophils, which generally rapidly penetrate the defective nerve, are a sufficient origin of TIMP-1-free MMP-9. In the neutrophil granules, TIMP-1-free MMP-9 coincides with NGAL. NGAL directly binds with MMP-9 and, as a consequence modulates MMP-9 activity protecting it from rapid degradation and self-destruction.^25,26^ Based on the important role of MMPs in vascular remodelling and their increased expression and activation under inflammatory and oxidative stress conditions, many studies have shown MMP imbalance to be a key event in atherosclerosis, arterial aneurysmal formation and plaque instability.^27^ Increased levels of TIMP-1 has been previously suggested as independent risk factors for hypertension, atherosclerosis and albuminuria, primarily in the early stages of diabetic nephropathy in type 2 diabetes to the progression of chronic kidney disease.^28^

Besides, TIMP-1 has been suggested as a marker for abnormal regulation of matrix remodelling after a cardiovascular event in patients with type 1 diabetes.^10^ Moreover, elevated TIMP-1 plasma levels have been associated with the presence of left ventricular hypertrophy, of heart failure with signs of hypertrophy and myocardial fibrosis and dysfunction.^29^ Our study demonstrated significant correlations between TIMP-1 and impaired compound muscle action potential (CMAP) amplitude and sensory nerve action potential (SNAP). Increased values of this biomarker associated with high neuropathy symptom assessment score as well as Neuropathy Impairment Assessment (NIA) scores. Besides, TIMP-1 is earlier reported to correlate with known factors of structural renal injury such as microalbuminuria and increased values of Cystatin C and NGAL.^30^ It was found that increased values of TIMP-1 were associated with decreasing eGFR, remaining in normal renal function range, showing its predictive role in early renal dysfunction. Thus, multiple regression analysis showed a significant predictive role of TIMP-1 in diagnosing early diabetic neuropathy. The same analysis showed that TIMP-1 along with NGAL might predict renal function impairment in patients with type 1 diabetes. Increased levels of MMP-9 alone or in parallel with its inhibitor TIMP-1 were found to associate with impaired NIA scores and abnormal heat and vibratory perception. Since it was previously shown that altered plasma levels of TIMP-1 and MMP-9 might associate with neuronal injury, their predictive value in diabetic nerve dysfunction may now be demonstrated. These markers have been associated mostly with inflammation, fibrosis and atherosclerosis making the theory of atherosclerosis behind the pathophysiology of diabetic microvascular complications.

Diabetic complications in type 1 diabetes might be devastating since they occur in young and productive ages. Thus, it is crucial to define predictive non-invasive methods and biomarkers for early microvascular complications to prevent or delay the start of irreversible long-term unfavourable outcomes and to minimise the rates of severe morbidity and mortality in these patients. A limitation of the current study is the small number of included patients and complications in this group, making it difficult to draw hard conclusions. However, results from small studies like this, could lead to better designed larger studies, where predictive markers can be further validated prior to their clinical use for screening and monitoring patients with type 1 diabetes.
